# Interleukin-18 Increases TLR4 and Mannose Receptor Expression and Modulates Cytokine Production in Human Monocytes

**DOI:** 10.1155/2015/236839

**Published:** 2015-03-19

**Authors:** Luciane Alarcão Dias-Melicio, Reginaldo Keller Fernandes, Daniela Ramos Rodrigues, Marjorie Assis Golim, Angela Maria Victoriano Campos Soares

**Affiliations:** ^1^Departamento de Patologia, Faculdade de Medicina, Universidade Estadual Paulista (UNESP), Campus Botucatu, 18618-970 Botucatu, SP, Brazil; ^2^Departamento de Microbiologia e Imunologia, Instituto de Biociências, Universidade Estadual Paulista (UNESP), Campus Botucatu, 18618-970 Botucatu, SP, Brazil; ^3^Faculdade de Medicina, Universidade Estadual Paulista (UNESP), Campus Botucatu, Hemocentro, 18618-970 Botucatu, SP, Brazil

## Abstract

Interleukin-18 is a proinflammatory cytokine belonging to the interleukin-1 family of cytokines. This cytokine exerts many unique biological and immunological effects. To explore the role of IL-18 in inflammatory innate immune responses, we investigated its impact on expression of two toll-like receptors (TLR2 and TLR4) and mannose receptor (MR) by human peripheral blood monocytes and its effect on TNF-*α*, IL-12, IL-15, and IL-10 production. Monocytes from healthy donors were stimulated or not with IL-18 for 18 h, and then the TLR2, TLR4, and MR expression and intracellular TNF-*α*, IL-12, and IL-10 production were assessed by flow cytometry and the levels of TNF-*α*, IL-12, IL-15, and IL-10 in culture supernatants were measured by ELISA. IL-18 treatment was able to increase TLR4 and MR expression by monocytes. The production of TNF-*α* and IL-10 was also increased by cytokine treatment. However, IL-18 was unable to induce neither IL-12 nor IL-15 production by these cells. Taken together, these results show an important role of IL-18 on the early phase of inflammatory response by promoting the expression of some pattern recognition receptors (PRRs) that are important during the microbe recognition phase and by inducing some important cytokines such as TNF-*α* and IL-10.

## 1. Introduction

Interleukin-18 (IL-18) belongs to the fourth member of the IL-1 family and is produced by a wide variety of cells including macrophages, dendritic cells (DCs), neutrophils, adipocytes, Kupffer cells, microglial cells, and certain neurons in the brain. This cytokine presents many unique biological effects, including pleiotropic, multifunctional, and proinflammatory actions [1–4].

Like IL-1*β*, the prototype member of the family, IL-18 secretion does not happen via endoplasmic reticulum and Golgi apparatus. The cytokine is produced as a leaderless and biologically inactive 24 kDa precursor protein called pro-IL-18, which is cleaved by IL-1*β* converting enzyme, called caspase-1, to produce 18 kDa mature and biologically active cytokine [3, 5, 6]. Caspase-1 is presented in an inactive 45 kDa precursor form whose activation requires assembly of multiunit complexes involving certain nucleotide-binding and oligomerization domain- (NOD-) like proteins, called inflammasomes, that are responsible for recruiting and activating caspase-1 precursor molecules [7–9]. So, an increased production of biologically active IL-18 requires two distinct stimuli: one increases IL-18 gene expression at mRNA and protein levels and usually comes from recognition of pathogen products by a pattern recognition receptor (PRR); the second signal causes inflammasome assembly, caspase-1 activation, and secretion of mature IL-18 [10–12].

IL-18 was initially described as an IFN-*γ*-inducing factor that upregulates the IL-12R*β* subunit on T cells and has generally been considered a Th1 type cytokine [13, 14]. However, depending on the context of stimulation, the cytokine microenvironment, and genetic predisposition, IL-18 can promote a Th1 or Th2 response [15]. The IL-18R*α* membrane protein is responsible for ligand binding and TIR domains present in the cytoplasmic tails of the receptor chains transduce signals in target cells, which involves MyD88- and TRAF6-dependent pathways to activate NF-*κ*B and JNK cascades [1, 2, 4, 16]. Although the pleiotropic effects of IL-18 show an important role in the modulation of Th2 cytokines, when acting independently of the action of IL-12 [15], its main function would be to participate in inflammatory response by inducing production of several proinflammatory cytokines and chemokines including TNF-*α*, IL-8, IL-1*β*, MIP-1*α*, NO, MMP, CXCL8, CXCL9, and CXXL10 from a variety of human cells [3, 4, 17].

The innate immune response is initiated through the activation of pattern recognition receptors (PRRs) by pattern-associated molecular patterns or PAMPs and endogenous molecules produced by injured tissue. These receptors regulate many aspects of innate immunity and determine the polarization and function of adaptive immunity [18–21], but they are also involved in the maintenance of tissue homeostasis by regulating tissue repair and regeneration [10, 20, 22]. TLRs are the most extensively studied recognition sensors that participate in the initiation of inflammation [20]. TLR2 recognizes peptidoglycan and lipoteichoic acids of Gram-positive bacteria. Besides, TLR2 is involved in the recognition of other bacterial components such as lipoprotein/lipopeptides, lipoarabinomannan, phenol-soluble modulin, porins, and glycolipids [23]. However, TLR4 can recognize lipopolysaccharide, heat shock proteins, flavolipin, mannan, fibrinogen, taxol, glycoinositolphospholipids, retroviral envelope protein, hyaluronic acid, and fibronectin [24].

Tissue-resident macrophages express all TLRs (except TLR3) and are highly responsive to their agonist [20]. In these cells, TLRs are important for each stage of phagocytosis, ranging from engulfment of invading pathogens to antigen processing and presentation of antigenic peptides. TLRs also lead to the production of cytokines such as tumor necrosis factor- (TNF-) *α* and interleukin- (IL-) 1*β*, and to the release of chemokines that induce endothelial cell activation and drive inflammatory cell recruitment, regulate the generation of vasoactive lipids and reactive oxygen species [20, 21, 25, 26]. In addition, TLR activation regulates the expression of major histocompatibility complex (MHC) molecules and costimulatory molecules [27] and induces the release of IL-12 and IL-10, cytokines which differentially alert DCs to polarize naive T cells and activate specific adaptive immunity [28].

The mannose receptor (MR, CD206) is a member of the MR family, which is a subgroup of the C-type lectin superfamily that comprises transmembrane and soluble proteins such as selectins and collectins and can bind terminal mannose, fucose, or N-acetyl glucosamine and consequently recognizes a wide variety of ligands, including several bacterial, viral, and fungal pathogens [29]. Thus, MR is considered a PRR and pathogens recognized by this receptor include* Candida albicans*,* Leishmania*,* Mycobacterium tuberculosis*, HIV,* Pneumocystis carinii*, dengue virus, and selected strains of* Klebsiella pneumonia*,* Cryptococcus neoformans,* and* Streptococcus pneumoniae* [29].

Despite its role on resistance of infections, high levels of IL-18 has been related to the pathogenesis of several disorders and diseases, such as Chronic kidney disease (CKD) [30], Atherosclerosis [31–33], Sickle cell anemia (SCA) [34], Acute Myocardial Infarction and Heart Failure [35–37], polycystic ovary syndrome [38], Severe Traumatic Brain Injury [39]; Chronic Obstructive Pulmonary Disease [40], hepatitis C [41], Autoimmune Hepatitis [42], and mainly sepsis, due Melioidosis, an infection caused by the gram-negative bacillus Burkholderia pseudomallei (formerly Pseudomonas) [43]. Therefore, the effect of IL-18 on PRRs expression and cytokines production could account for the severity of the inflammatory response observed in these diseases, particularly in sepsis.

Thus, the present study was designed to better elucidate the role of IL-18 on the expression of some PRRs such as TLR2, TLR4, and MR by human monocytes isolated from peripheral blood, and its effect on TNF-*α*, IL-12, IL-15, and IL-10 production by these cells, once IL-18 is involved in the development of various diseases as mentioned above. The results presented herein demonstrate a clear role of IL-18 in directly modulating TLR4 and MR expression and TNF-*α* and IL-10 production by these cells.

## 2. Subjects and Methods

### 2.1. Donors

Fifteen healthy blood donors from the Faculdade de Medicina de Botucatu (FMB), UNESP, Brasil (age range 20–50 years), were included in this study. The Research Ethics Committee approved the study, and informed consent was obtained from all the subjects (2513/07).

### 2.2. Monocyte Isolation

Heparinized venous blood was obtained from healthy adults. Peripheral blood mononuclear cells (PBMC) were isolated by density gradient centrifugation at 400 g for 30 min on Ficoll-Paque Plus (density (*d*) = 1.077) (GE Healthcare Bio-Sciences AB, Uppsala). Briefly, heparinized blood was mixed with an equal volume of RPMI-1640 tissue culture medium (Sigma-Aldrich, St. Louis, USA), and samples were layered over 10 mL of Ficoll-Paque Plus in a 50 mL conical plastic centrifuge tube. After centrifugation at 400 g for 30 min at room temperature, the interface layer of PBMC was harvested and washed twice with RPMI-1640 tissue culture medium (Sigma-Aldrich). The PBMC suspension was stained with neutral red (0.02%) which is incorporated by monocytes and allows their identification and counting in a hemocytometer chamber. After counting, the mononuclear cell suspension was adjusted to 1 × 10^6^ monocytes/mL in RPMI-1640 (Sigma-Aldrich) containing 10% heat-inactivated fetal calf serum (Complete Tissue Culture Medium—CTCM), dispensed into 1000 *μ*L/well in 24-well flat-bottom plates (TPP, Trasadingen, Switzerland) and used for flow cytometry analysis and for cytokine production. After incubation of cultures for 1 h at 37°C in 5% CO_2_, nonadherent cells were removed by aspiration and each well was rinsed twice with RPMI-1640. This procedure resulted in cultures with more than 95% of monocytes. The resulting monocyte cultures were treated or not with IL-18 (MBL, Medical & Biological Laboratories Co. Ltda), 100 ng/mL, for 18 h at 37°C in 5% CO_2_. In some cocultures, anti-IL-18 (MBL), 0.5 *μ*g/mL, was used before the IL-18 treatment, to block IL-18 effects. Control groups with negative isotype control were also tested.

### 2.3. Flow Cytometry

For CD14, TLR2, TLR4, and MR expression, adherent monocytes were detached from wells by putting the plate on ice and using HyQTase Cell Detachment Solution (HyClone Laboratories Inc., Logan, UT, USA). After that, cells were put into polystyrene tubes for cytometric analysis (BD Labware, Franklin Lakes, NJ USA) and were washed and incubated with mouse anti-human CD14-PE/Cy7, mouse anti-human CD206-APC (MR), mouse anti-human TLR2-FITC, and mouse anti-human TLR4-PE (all from BioLegend, Inc., San Diego, CA) according to the instructions of the manufacturer. Nonspecific signals were calculated and attenuated by isotype control (BioLegend) tubes. After incubation for 20 min at room temperature in the dark, cells were washed and a fixative solution consisting of 5% formaldehyde in buffer (Becton Dickinson, San Jose, CA) was added; then cells were analyzed. Control experiments showed that HyQTase Cell Detachment Solution did not affect cell viability nor altered the expression of all receptors evaluated (data not shown).

For TNF-*α*, IL-10, and IL-12 intracellular analyses, monocyte cultures were pretreated with Brefeldin A Solution (BioLegend), six hours prior to harvest. Afterwards, detached monocytes were distributed into polystyrene tubes for cytometric analysis (BD Labware). Cells were washed and incubated with mouse anti-human CD14-PE/Cy7 (BioLegend), according to the manufacturer's instructions. Next, the permeabilization and staining procedures were conducted using a Cell Permeabilization Kit FIX&PERM (ADG, AN DER GRUB Bio Research GMBH, Kaumberg, Austria). Cells were stained with rat anti-human IL-10-PE, mouse anti-human IL-12/IL-23 p40-FITC, and mouse anti-human TNF-*α*-APC (all from BioLegend). Nonspecific signals were calculated and attenuated by isotype control (BioLegend) tubes. After incubation for 20 min at room temperature in the dark, cells were washed and a fixative solution consisting of 5% formaldehyde in buffer (Becton Dickinson) was added; then cells were analyzed.

For both, cells were analyzed with a FACSCalibur flow cytometer (Becton Dickinson). Data (an average of 10,000 events per sample) were analyzed with the software CELL QUEST (Cell Quest Software).

### 2.4. Measurement of Cytokines

After IL-18 treatment, monocyte culture supernatants were separated from cell debris by centrifugation at 1000 g for 15 min and stored at −80°C. The TNF-*α*, IL-10, IL-12, and IL-15 concentrations were measured by capture ELISA using BD OptEIA human ELISA Set (BD Biosciences, Franklin Lakes, NJ, USA). IL-18 concentrations were measured by human IL-18 ELISA Kit (MBL). ELISA was performed according to the manufacturer's protocols. Cytokine concentrations were determined with reference to a standard curve for serial twofold dilutions of recombinant cytokines. Absorbance values were measured at 492 nm using a micro-ELISA reader (MD 5000; Dynatech Laboratories).

### 2.5. Statistical Analysis

Data were analyzed statistically using GraphPad Prism software (GraphPad Prism 5.0, San Diego, CA). The results were compared by Friedman test, followed by Dunn's Multiple Comparison Test, with the significance level set at *P* < 0.05.

## 3. Results and Discussion

The innate immune system promptly responds to the invasion of microbes and acts as the first line of defense, whereby innate immune cells such as macrophages or DCs play a central role in the production of proinflammatory cytokines and nitric oxide after recognition of pathogen [44]. This response is triggered by PRRs that interact with pathogen structures and send signals to the host cell.

**Figure 1:**
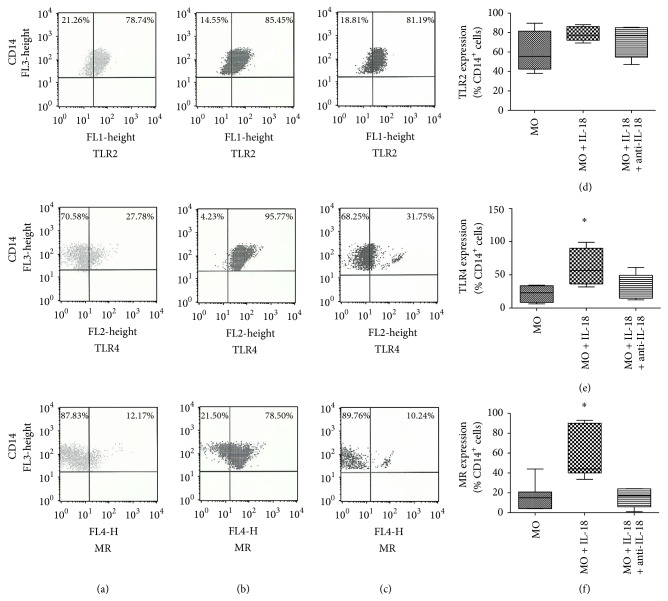
TLR2, TLR4 and MR expression in CD14^+^ monocytes. CD14^+^ monocytes (column (a)) were treated with IL-18 (100 ng/mL) (column (b)), or IL-18 (100 ng/mL) plus anti-IL-18 (0,5 *μ*g/mL) (column (c)), for 18 hours and evaluated by flow cytometry. Box-and-whisker plot showing data distribution of 15 healthy subjects tested for TLR2 (d), TLR4 (e), and MR (f). Horizontal lines represent the median values; boxes represent the 25th to 75th percentiles and vertical lines the 10th to 90th percentiles. ^*^Statistical significance between groups is indicated (*P* < 0.05  × other groups).

To better understand the role of IL-18 in the expression of TLR2, TLR4, and MR by purified CD14^+^ monocytes, cells were treated with IL-18 and subsequently analyzed by flow cytometry. The results showed that IL-18 was able to increase TLR4 and MR expression by CD14^+^ monocytes (Figure 1). However, the cytokine treatment did not affect TLR2 expression (Figure 1(d)). The blocking of IL-18 with specific neutralizing antibody showed a reversal on the TLR4 and MR expression results, as shown in Figures 1(e) and 1(f). The treatment with negative isotype control did not affect the response of monocytes (data not shown). These are new data that support the autocrine role of IL-18 by identifying an important direct modulation of TLR4 and MR on human monocytes by this cytokine, seeing that purified human monocytes treated with this cytokine presented higher expression of TLR4 and MR than control cells, whereas the blocking of IL-18 with anti-IL18 reversed this effect.

TLR-2 and TLR-4 are constitutively expressed by various cell members of the immune system including macrophages, neutrophils, and DCs (reviewed [20]). The expression of TLR-2 and TLR-4 is tightly regulated by several proinflammatory cytokines. But until now, the role of IL-18 in the expression of PRRs is not completely understood, and studies have reported an indirect effect of IL-18 on these cells via Th1 activation. Radstake et al. [45] showed that TLR-2 and TLR-4 are expressed in synovial tissue of patients with rheumatoid arthritis, with clinically active disease, and these expressions were associated with the levels of both IL-12 and IL-18. However, IL-12 and IL-18 treatment* in vitro* did not affect the expression of TLR-2 or TLR-4 on purified monocytes. An upregulation of TLR-2 and TLR-4 was just seen when PBMC were treated with IL-18. This effect was inhibited by the blocking of IFN-*γ*, thus showing an indirect role of IL-18 on TLR2 and TLR4 expression via induction of IFN-*γ* by T cells [45].

This increased expression of TLR4 by IL-18 that was detected in this study could promote a series of events, after pathogen recognition, trigging the production of cytokines. After ligand binding, TLRs dimerize and undergo conformational changes, which are required for the recruitment of adaptor molecules, via their TIR domains. These adaptor molecules, namely, MyD88, Mal (MyD88 adapter-like)/TIRAP (TIR-domain-containing adaptor protein), TRIF (Toll-receptor-associated activator of interferon), TRAM (TRIF-related adaptor molecule), and SARM (sterile *α* and armadillo motifs), contribute to the specificity of individual responses to pathogens. Each TLR can mediate a tailored response in association with different combinations of these adaptors. Two major pathways can be activated by TLRs; the MyD88-dependent pathway results in the activation of NF-*κ*B and activating protein-1 (AP-1), regulating the transcription, mRNA stability, and translation of numerous proinflammatory cytokine genes, such as TNF-*α*, IL-6, IL-12, and IFNs, while the TRIF-dependent pathway results in the activation of type I interferons (IFNs) [46].

Thus, this direct effect of IL-18 on the increase of TLR4 expression could account on several inflammatory diseases. One of the most important disease, that presents high levels of IL-18 (more than 10,000 pg/mL) is sepsis [43]. It was observed that patients with severe Gram-negative infection (Melioidosis) had elevated levels of IFN-*γ*, IL-18, IL-12p40, and IL-15 on admission, with significantly higher levels in blood culture-positive [43]. It was also observed that IFN-*γ* production by whole blood stimulated with heat-killed* Burkholderia pseudomallei* was inhibited by anti-IL-12 treatment more than anti-IL-18 or anti-IL-15, and the effect of anti-IL-12 was further enhanced by anti-IL-18 treatment, suggesting that, during Gram-negative sepsis, IFN-*γ* production is controlled at least in part by endogenous IL-18, IL-12, and IL-15 [43]. Puren et al. [47] in a previous study evaluated a simple 24 h human whole blood culture that was treated with IL-18 in different concentrations, plus low concentration of LPS, showing that only IL-18 did not induce IFN-*γ* production. However, the combination of LPS plus increasing concentrations of IL-18 (0.625–10 nM) resulted in an increased IFN-*γ* production in a dependant manner. The combination, however, was independent of the concentration of LPS. It was also detected that cultures treated with IL-18 + LPS showed an increased production of IL-6, IL-8, and TNF-*α*, and LPS-induced TNF-*α* production was potentiated by IL-18 [47]. Recently, it was also demonstrated that the exposure of RAW264.7 cells to LPS/ATP triggered the activation of caspase-1 and the cleavage of interleukin- (IL-) 1*β*, as well as the release of other cytokines, such as IL-18 and IL-33 [48]. Thus, once demonstrated that LPS/ATP triggered the activation of caspase-1 with release of IL-18 [48], as well as the identification of IL-18 effect on TLR4 expression, this could also explain the systemic activation of cells, and amplification of the response observed in sepsis.

Another important PRR is the MR (CD206), a type I transmembrane protein that possesses eight extracellular CTLDs and a short cytoplasmic tail which lacks classical signaling motifs; it is expressed by macrophages, some DCs, and a variety of other cells and tissues [29, 49, 50]. The MR has been shown to induce a variety of cellular responses, but the molecular mechanisms responsible for transducing the intracellular signals from this receptor are unclear. The recognition of microorganisms by this receptor has been shown to promote the production of a number of cytokines such as TNF-*α*, GM-CSF, IL-12, IL-8, IL-6, and IL-1*β*, although there is also evidence that the MR can inhibit the production of certain cytokines, including TNF-*α* [29, 50–52]. A mechanism that could account for the negative effect of MR ligation on proinflammatory cytokine production is the upregulation of IRAK-M (an inhibitor of TLR signaling that blocks the dissociation of IRAK-1 and IRAK-4 from MyD88), since this regulator could be induced by treatment with the MR ligand mannan [53]. Rajaram et al. [54] reported that virulent* Mycobacterium tuberculosis* and mannose-capped lipoarabinomannan induce the expression of nuclear receptor/transcriptional factor PPAR*γ* (peroxisome proliferator-activated receptor *γ*) in human macrophages and that this upregulation of PPAR*γ* expression was mediated by the MR. The induction of this new pathway serves as a negative regulator of macrophage activation by altering the expression of many inflammatory genes [54–56], modulating macrophage differentiation and activation through transrepression of the transcription factors NF-*κ*B, AP-1, and STAT [57–61], and attenuating the respiratory burst [62]. These attributes have important implications for the control of infections. But although the MR plays a clear role in homeostasis, its role in antimicrobial immunity remains unclear [50]. Besides that, MR has been considered an important marker of M2 macrophages, mainly in adipose tissue macrophages (ATMs) [63], and recent studies showed increase of IL-18 expression in subcutaneous and abdominal adipose tissues of obese subjects or with metabolic syndrome, and in monocyte-derived macrophage cultures exposed to hyperglycaemia [64, 65]. Study also showed that circulating levels of IL-18 were higher in obese subjects [64]. Now, it was showed that leptin stimulates caspase-1 activity in monocytes and that leptin-induced IL-18 secretion is dependent on caspase-1 activity suggesting a signalling pathway between leptin and the inflammasome in these cells [66]. The authors suggested that leptin-stimulated IL-18 could be explained by a secondary effect of the upregulation of other cytokines, such as TNF-*α*, as having been described by other studies [67], once leptin had no direct effect on monocyte TNF-*α* secretion [68]. Confirming these results, Esser et al. [69] showed that the metabolically unhealthy obese phenotype seems to be associated with an increased activation of the NLPR3 inflammasome in macrophages infiltrating visceral adipose tissue. Thus, our results suggest that IL-18 production could account for MR expression and induce ATMs into alternative M2 macrophages.

**Figure 2:**
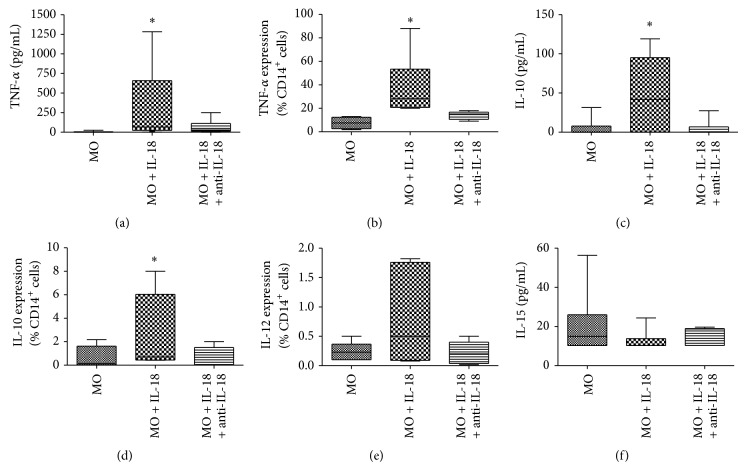
Production of TNF-*α* ((a) and (b)), IL-10 ((c) and (d)), IL-12 (e), and IL-15 (f) in CD14^+^ monocytes treated with IL-18 (100 ng/mL) or IL-18 (100 ng/mL) plus anti-IL-18 (0,5 *μ*g/mL), for 18 hours in culture supernatants, evaluated by ELISA ((a), (c), and (f)), and intracellular staining, by flow cytometry ((b), (d), and (e)). Box-and-whisker plot showing data distribution of 15 healthy subjects tested. Horizontal lines represent the median values; boxes represent the 25th to 75th percentiles and vertical lines the 10th to 90th percentiles. ^*^Statistical significance between groups is indicated (*P* < 0.05  × other groups).

Knowing the ability of IL-18 to induce either Th1 or Th2 responses [15], we also tested the capacity of IL-18 to induce some pro- or anti-inflammatory cytokines by CD14^+^ monocytes. The IL-18 treatment induced an increase in TNF-*α* (Figures 2(a) and 2(b)) and IL-10 (Figures 2(c) and 2(d)) levels by CD14^+^ monocytes. The productions of intracellular IL-12 (Figure 2(e)) and IL-15 (Figure 2(f)) were not induced by IL-18. The IL-12 levels in culture supernatant of CD14^+^ monocyte controls and in that treated with IL-18 were undetected by ELISA, while IL-15 levels did not differ between groups. The blocking of IL-18 with specific neutralizing antibody reversed the effect of IL-18 on TNF-*α* and IL-10 production by these cells. The treatment with negative isotype control did not affect the production of the quantified cytokines (data not shown). Then, our results showed an important role of IL-18 in increasing the endogenous production of IL-10 and TNF-*α* by monocytes.

TNF-*α* is known to induce proinflammatory activities through various cell types including mononuclear and polymorphonuclear phagocytes, in which it is responsible for the activation of cytocidal systems and plays a major role in host defense [70–72]. Takahashi et al. [73] demonstrated the increased production of TNF-alpha, IL-12, and IFN-*γ* by PBMC treated with IL-18. These results were correlated with the upregulation of ICAM-1, B7.2, and CD40 expression on monocytes. Blocking the engagement of these adhesion molecules by antibodies against ICAM-1 and B7.2 reduced the cytokine production by IL-18-treated PBMC [74, 75]. The authors suggested that IL-18 induces cytokine production through upregulation of adhesion molecule expression on monocytes [73]. But now, our results show a direct effect of IL-18 on purified monocytes by inducing TNF-*α* production that together with effects shown on the TLR4 receptor could further compromise the response mainly in sepsis.

On the contrary, IL-10 is a cytokine produced by CD4^+^ T helper type 2 (TH_2_) cells, CD8^+^ T cells, monocytes, macrophages, and B cells. It was first described as an inhibitor of activation and cytokine production by TH_1_ cells [76]. However, IL-10 suppresses the activity of T and NK cells indirectly, via monocyte and macrophage inhibition, and is considered a macrophage deactivation factor [76]. This IL-10 effect may occur mainly by influencing macrophage recruitment, viability, morphology, phagocytosis, the production of cytokines and expression of their receptors such as the major histocompatibility complex and costimulatory molecules, antigen presentation, generation of reactive oxygen and nitrogen intermediates, and the killing of microbes and tumor cells [76, 77]. Studies suggest that IL-10, beyond acting on monocytes/macrophages and lymphocytes, may also exert an important regulatory action on neutrophil functions [78, 79].

We would like to point out that, in this study, these new very interesting results regarding the direct effect of IL-18 on human monocytes by inducing both TNF-*α* and IL-10 production and MR expression could indicate that IL-18 participates on the induction of both classically (M1) and alternatively (M2) activated macrophages, once after IL-18 treatment, cultured cells presented higher TNF-*α*, IL-10 production and MR expression. M1 macrophages are characterized by high microbicidal capacity and secretion of proinflammatory cytokines such as TNF-*α*, while M2 macrophages present high expression of mannose, galactose, and scavenging receptors, more phagocytic activity, and a phenotype characterized by high expression of IL-10 and low expression of IL-12 [80, 81]. Further investigations are being conducted in our laboratory to better elucidate these mechanisms.

## 4. Conclusions

In conclusion, our findings showed that IL-18 affects TLR4 and MR expression on human monocytes, and TNF-*α* and IL-10 production by these cells. Taken together, these results implies that this cytokine may also play an important role in the initiation of innate immune responses, participating in severity or resolution of infections and inflammatory diseases, since monocytes and macrophages are the main components of this response.
